# Embolism resistance in stems of herbaceous Brassicaceae and Asteraceae is linked to differences in woodiness and precipitation

**DOI:** 10.1093/aob/mcy233

**Published:** 2018-12-24

**Authors:** Larissa Chacon Dória, Cynthia Meijs, Diego Sotto Podadera, Marcelino Del Arco, Erik Smets, Sylvain Delzon, Frederic Lens

**Affiliations:** 1 Naturalis Biodiversity Center, Leiden University, RA Leiden, The Netherlands; 2 Programa de Pós-Graduação em Ecologia, UNICAMP, Campinas, São Paulo, Brazil; 3 Department of Plant Biology (Botany), La Laguna University, La Laguna, Tenerife, Spain; 4 BIOGECO INRA, Université Bordeaux, Pessac, France

**Keywords:** Canary Islands, drought, embolism resistance, herbaceous species, stem anatomy, thickness of intervessel pit membranes, woodiness, xylem hydraulics

## Abstract

**Background and Aims:**

Plant survival under extreme drought events has been associated with xylem vulnerability to embolism (the disruption of water transport due to air bubbles in conduits). Despite the ecological and economic importance of herbaceous species, studies focusing on hydraulic failure in herbs remain scarce. Here, we assess the vulnerability to embolism and anatomical adaptations in stems of seven herbaceous Brassicaceae species occurring in different vegetation zones of the island of Tenerife, Canary Islands, and merged them with a similar hydraulic–anatomical data set for herbaceous Asteraceae from Tenerife.

**Methods:**

Measurements of vulnerability to xylem embolism using the *in situ* flow centrifuge technique along with light and transmission electron microscope observations were performed in stems of the herbaceous species. We also assessed the link between embolism resistance vs. mean annual precipitation and anatomical stem characters.

**Key Results:**

The herbaceous species show a 2-fold variation in stem *P*_50_ from –2.1 MPa to –4.9 MPa. Within *Hirschfeldia incana* and *Sisymbrium orientale*, there is also a significant stem *P*_50_ difference between populations growing in contrasting environments. Variation in stem *P*_50_ is mainly explained by mean annual precipitation as well as by the variation in the degree of woodiness (calculated as the proportion of lignified area per total stem area) and to a lesser extent by the thickness of intervessel pit membranes. Moreover, mean annual precipitation explains the total variance in embolism resistance and stem anatomical traits.

**Conclusions:**

The degree of woodiness and thickness of intervessel pit membranes are good predictors of embolism resistance in the herbaceous Brassicaceae and Asteraceae species studied. Differences in mean annual precipitation across the sampling sites affect embolism resistance and stem anatomical characters, both being important characters determining survival and distribution of the herbaceous eudicots.

## INTRODUCTION

Hydraulic failure is one of the main physiological mechanisms associated with reductions in forest productivity and drought-induced tree mortality (Choat *et al.*, 2012; Anderegg *et al.*, 2016; Adams *et al.*, 2017). Water movement inside the conduits is prone to dysfunction due to negative xylem pressures generating metastable conditions (Tyree and Sperry, 1989; Tyree and Zimmermann, 2002). With increasing drought stress, embolisms could propagate from a gas-filled conduit to a neighbouring functional conduit through interconduit pit membranes, potentially generating lethal levels of embolisms (Tyree and Zimmermann, 2002; Brodribb *et al.*, 2010; Brodersen *et al.*, 2013). The vulnerability to xylem embolism can be measured by vulnerability curves, in which the percentage loss of hydraulic conductivity is plotted against the xylem pressure (Cochard *et al*., 2010, 2013). The *P*_50_ value, referring to the negative pressure associated with 50 % loss of hydraulic conductivity, is an oft-cited proxy for plant drought resistance, although it does not present a critical threshold value for angiosperms (Urli *et al.*, 2013; Adams *et al.*, 2017).

There is considerable interspecific variation in *P*_50_ across plant species, from –0.5 MPa up to –19 MPa, and the majority of studies show that species from dry environments are generally more resistant to embolism (more negative *P*_50_) than species from wet environments (Choat *et al.*, 2012; Lens *et al*., 2013, 2016; Larter *et al.*, 2015). Knowledge about intraspecific variation in *P*_50_ remains scarce and provides contradictory results: it seems to be species specific, but it can vary either considerably (Kolb and Sperry, 1999; Choat *et al.*, 2007; Corcuera *et al.*, 2011; Nolf *et al*., 2014, 2016; Volaire *et al.*, 2018; Cardoso *et al.*, 2018) or subtly (Holste *et al.*, 2006; Martínez-Vilalta *et al*., 2009; Lamy *et al*., 2013; Ahmad *et al*., 2017), or may even be absent (Maherali *et al.*, 2009; Wortemann *et al.*, 2011) for woody as well as for herbaceous species.

There is a vast body of literature available focusing on hydraulic conductivity and safety for hundreds of woody species (Maherali *et al.*, 2004; Pittermann *et al.*, 2010; Choat *et al.*, 2012; Bouche *et al.*, 2014; Gleason *et al.*, 2016). Herbs, on the other hand, remain poorly investigated: *P*_50_ values of stems are available for <30 species, of which a minority are eudicots while most species are grasses (e.g. Mencuccini and Comstock, 1999; Stiller and Sperry, 2002; Kocacinar and Sage, 2003; Holste *et al.*, 2006; Maherali *et al.*, 2009; Rosenthal *et al.*, 2010; Lens *et al*., 2013, 2016; Nolf *et al*., 2014, 2016; Skelton *et al.*, 2017; Dória *et al.*, 2018; Volaire *et al.*, 2018). Based on this limited data set, most herbaceous species studied so far are sensitive to embolism formation in their stems, with a *P*_50_ of around –2.5 MPa. However, some of the grass stems studied are remarkably resistant to embolism formation (up to –7.5 MPa), implying that both herbs and trees share the ability to support very negative water potentials without embolism formation during drought stress (Lens *et al.*, 2016).

In this study, we focus on the research field of xylem hydraulics in herbaceous stems which has been largely neglected, despite the overwhelming occurrence of economically important herbaceous food crops (Monfreda *et al.*, 2008) and the dependency on grazed grasslands for our livestock. The main reason for neglecting herb hydraulics is that their fragile stems and often low hydraulic conductance make vulnerability curves technically more challenging. However, recent fine-tuning of the high-throughput *in situ* flow centrifuge method (cavitron; Lens *et al.*, 2016; Dória *et al.*, 2018) and the new optical vulnerability technique (Skelton *et al.*, 2017) have yielded stem *P*_50_ data of herbaceous species, which opens up new opportunities to boost the virtually neglected aspect of herb hydraulics and predict future crop productivity and survival (Challinor *et al.*, 2009), especially in a world facing climate change (Rahmstorf and Coumou, 2012; Dai, 2013).

In addition to the understudied aspect of herb hydraulics, we also investigate stem anatomical characters to assess poorly known structure–function relationships in herbaceous stems. Plant sensitivity to drought-induced embolism is determined by a whole suite of stem anatomical characters in woody trees (Hacke and Jansen, 2009; Lens *et al.*, 2011; Jacobsen *et al.*, 2012; Pivovaroff *et al*., 2016; Pereira *et al.*, 2017; O’Brien *et al.*, 2017), of which the thickness of intervessel pit membranes is probably one of the most hydraulically relevant anatomical features, altering both water flow efficiency and the spread of potential lethal levels of embolism in the xylem (Jansen *et al.*, 2009; Lens *et al.*, 2011; Li *et al.*, 2016; Gleason *et al.*, 2016; Dória *et al.*, 2018). Furthermore, vessel diameter is an informative character determining xylem area-specific conductivity (*K*s) (Hacke *et al.*, 2016), but also correlates with plant height, environmental constraints and, potentially, embolism resistance (Davis *et al.*, 1999; Olson and Rosell, 2013; Schreiber *et al.*, 2015; Hacke *et al.*, 2016; Olson *et al.*, 2018). Mechanical characters such as wood density, total degree of lignification, thickness-to-span ratio of vessels and thickness of the intervessel wall have also been linked to increasing drought stress resistance (Hacke *et al.*, 2001; Jacobsen *et al*., 2005, 2007; Chave *et al.*, 2009; Hoffman *et al.*, 2011; Pratt and Jacobsen, 2017). These mechanical characters are often reported as indirectly linked to embolism resistance, since embolism formation and spread occur at the pit level (Bouche *et al.*, 2014; Pereira *et al.*, 2017; Dória *et al.*, 2018).

In herbaceous eudicots, an increase in embolism resistance is linked to an increase in wood formation, which reflects an increase in the proportion of lignified area per total stem area (Lens *et al*., 2013, 2016; Tixier *et al.*, 2013; Dória *et al.*, 2018), and also grasses that are more resistant to embolism formation have more lignified stems compared with the more vulnerable species (Lens *et al.*, 2016). Wood formation has been observed in many herbaceous eudicots, especially at the base of the stem, and several studies show a continuous range in the degree of wood formation between stems of herbaceous eudicot species (Dulin and Kirchoff, 2010; Schweingruber *et al.*, 2011; Lens *et al.*, 2012*a*; Kidner *et al.*, 2016; Dória *et al.*, 2018). This highlights the fuzzy boundaries between woodiness and herbaceousness, leading to intermediate life forms such as ‘woody herbs’ or ‘half shrubs’ (Lens *et al.*, 2012*a*), but species with these intermediate life forms do not form a wood cylinder that extends towards the upper parts of the stem and are therefore considered as herbaceous (Kidner *et al.*, 2016).

In this study, we combine hydraulic measurements with detailed stem anatomical characteristics and climatic variables (from meteorological stations near the sampling sites) to investigate structure–function relationships in stems of seven herbaceous species belonging to the Brassicaceae family from the island of Tenerife (Canary Islands, Spain), and merged this data set with a similar data set for four herbaceous Asteraceae species that were sampled on the same island for a previous publication (Dória *et al.*, 2018). The main reason for selecting Tenerife is the huge range of climatic conditions in a small area of 2034 km^2^, ranging from the humid northern laurel forests of Anaga to the dry southern desert-like region around El Médano, separated by the tall Teide volcano (approx. 3700 m asl) generating different altitudinal vegetation types (del-Arco *et al.*, 2006). We address the following questions. (1) Do herbaceous species growing in drier environments have more embolism-resistant stems, both across and within species? (2) What are the stem anatomical characters that explain the variation in embolism resistance amongst the species studied? (3) Is there any relationship between precipitation and both xylem vulnerability to embolism and anatomical characters?

## MATERIALS AND METHODS

### Plant material and climate data

We collected the Brassicaceae specimens throughout the island of Tenerife, in different vegetation zones with different mean annual precipitation and aridity indices. The climatic data of precipitation and temperature for each of the sampling sites were provided by Agencia Estatal de Meteorología (AEMET, Spanish Government), covering a period from 110 to 30 years depending on the meteorological station. We received the data from five different meteorological stations (Anaga San Andrés, Arico Bueno, Arafo, Laguna Instituto and Vilaflor) matching the five sampling sites (Supplementary Data Fig. S1). We used the mean annual precipitation for each site, and calculated the potential evapotranspiration using the Thornthwaite equation (1948). The aridity indices were calculated as a ratio of mean annual precipitation to mean annual potential evapotranspiration (UNEP, 1997). Since this aridity index is highly correlated with mean annual precipitation (*P* < 0.001, *r* = 0.993) we opted to select the former in the statistical models.

The collection trip was carried out in March 2017, matching with the wet, flowering period of the herbaceous species. We harvested seven annual Brassicaceae species: *Hirschfeldia incana* (L.) Lagr.-Fossat, *Raphanus raphanistrum* L., *Rapistrum rugosum* L. All., *Sinapis alba* L., *Sinapis arvensis* L., *Sisymbrium erysimoides* Desf. and *Sisymbrium orientale* L. The time of germination is similar for all species studied and it is linked to the arrival of the rains in autumn and winter. However, there can be small differences between populations, amongst and within species: populations growing on the northern slopes of the island generally germinate earlier than plants growing on the southern slopes due to the moist north-eastern trade winds, and populations from higher altitudes usually germinate later than plants from lower altitudes.

The specimens of *H. incana* and *S. orientale* were collected from two different populations occurring in contrasting environments. The northern area of La Laguna (mean annual precipitation = 526.9 mm; aridity index = 0.68) and the southern area of Vilaflor (mean annual precipitation = 396.3 mm; aridity index = 0.53) were the wetter collection sites for *H. incana* and *S. orientale* populations, respectively. The drier sites were the southern areas of Guímar (mean annual precipitation = 311.8 mm; aridity index = 0.39) and the region of Arico Bueno (mean annual precipitation = 264.3 mm; aridity index = 0.34), for *H. incana* and *S. orientale*, respectively (Supplementary Data Fig. S1).

The four annual species of Asteraceae, *Cladanthus mixtus* (L.) Oberpr. & Vogt., *Coleostephus myconis* (L.) Cass.*, Glebionis coronaria* (L.) Cass ex Spach and *Glebionis segetum* (L.) Fourr. included in this study were investigated by Dória *et al.* (2018), during the spring of 2016 in Tenerife in the area of La Laguna (mean annual precipitation = 526.9 mm; aridity index = 0.68), following the same methodological procedures described below. For both the Brassicaceae and Asteraceae species, we harvested 10–20 individuals per species. All the species studied are annual herbaceous species, but some species (especially *S. alba* and *S. arvensis*) show a tendency to become biannual, which may be a consequence of the release of seasonality compared with the European mainland (Carlquist, 1974).

All individuals were collected from the soil, with roots still attached, quickly wrapped in wet tissues and sealed in plastic bags. Afterwards, the stems were stored in a cold room (around 5 ºC) for a maximum of 5 d at the University of La Laguna, Tenerife. The sealed plastic bags were shipped by plane and immediately stored in a fridge for a maximum of 2 weeks at the caviplace facility to perform the hydraulic measurements (University of Bordeaux, France).

### Xylem vulnerability to embolism

One to three stems per individual from at least ten individuals per species were used to measure vulnerability to embolism. Prior to measurements, all the stems were cut under water in the lab with a razor blade into a standard length of 27 or 42 cm in order to fit the two cavitron rotors used, and we confirmed that the vessels were shorter than the stem segments using the air pressure technique at 0.2 MPa. The cavitron is a modified centrifuge allowing the negative pressure in the central part of the stem segment to be lowered by spinning the stems at different speeds while simultaneously measuring the water transport in the vascular system (Cochard, 2002; Cochard *et al.*, 2013). First, the maximum hydraulic conductance of the stem in its native state (*K*_max_ in m^2^ MPa^–1^ s^–1^) was calculated under xylem pressure close to zero MPa using a reference ionic solution of 10 mm KCl and 1 mm CaCl_2_ in deionized ultrapure water. The rotation speed of the centrifuge was then gradually increased by –0.5 or –1 MPa to lower xylem pressure. The percentage loss of conductivity (PLC) of the stem was determined at each pressure step following the equation:

$$\text{PLC}=100\left(1-\frac{K}{K_{\text{max}}}\right)$$ (1)

where *K*_max_ represents the maximum conductance of the stem and *K* represents the conductance associated at each pressure step.

The vulnerability curves, showing the change in percentage loss of conductivity according to the xylem pressure, were obtained using the Cavisoft software (Cavisoft v1.5, University of Bordeaux, Bordeaux, France). A sigmoid function (Pammenter and Van der Willigen, 1998) was fitted to the data from each sample, using the following equation with SAS 9.4 (SAS 9.4, SAS Institute, Cary, NC, USA):

$$\text{PLC}=\;\frac{100}{\left[1+\exp\left(\frac{S}{25}*\;\left(P_{i}-P_{50}\right)\right)\right]}$$ (2)

where *S* (% MPa^–1^) is the slope of the vulnerability curve at the inflexion point, *P* is the xylem pressure value used at each step, and *P*_50_ is the xylem pressure inducing 50 % loss of hydraulic conductivity. The parameters *S* and *P*_50_ were averaged for each species.

### Stem anatomy

Light microscopy (LM), scanning electron microscopy (SEM) and transmission electron microscopy (TEM) were performed at Naturalis Biodiversity Center, the Netherlands, based on the samples for which we had obtained suitable vulnerability curves. The samples were taken from three individuals per species for LM and SEM, and from two individuals per species for TEM, from the middle part of the stem, where the negative pressure caused embolism formation during the cavitron experiment. The lab protocols for LM, SEM and TEM followed Dória *et al.* (2018). All the anatomical measurements were done using ImageJ (National Institutes of Health, Bethesda, MD, USA), largely following the suggestions of Scholz *et al.* (2013) and the IAWA Committee (1989).

Amongst the anatomical characters measured using LM, several indicators for lignification were calculated using a cross-section, such as the proportion of lignified area per total stem area [P_LIG_, measuring the sum of primary xylem area, secondary xylem (= wood) area and fibre caps area in the cortex and dividing it by the total stem area], the proportion of xylem fibre wall area per fibre area (P_FW_F_X_, at the level of a single cell), and the thickness-to-span ratio of vessels (T_W_D_V_). The diameter of vessels (D_V_) was calculated based on the lumen area that was considered to be a circle according to the equation:

$$DV=\;\sqrt{\frac{4A}{\pi }}$$ (3)

where D_V_ is the vessel diameter and A is the vessel lumen area. The hydraulically weighted vessel diameter (D_H_) was calculated following the equation:

$$DH=\;\frac{\sum^{\text{​}}DV^{5}}{\sum^{\text{​}}DV^{4}}$$ (4)

where D_V_ is the vessel diameter as measured in eqn (3).

The ultrastructure of intervessel pits was observed using a field emission scanning electron microscope (Jeol JSM-7600F, Tokyo, Japan) and a JEOL JEM 1400-Plus transmission electron microscope (JEOL, Tokyo, Japan), as described in Dória *et al.* (2018). Since we observed intervessel pit membranes from the central stem segment parts where centrifugal force was applied, our measurements provide a relative estimation of intervessel pit membrane thickness.

### Statistical analyses

We tested the effect of both species and mean annual precipitation on the various hydraulic parameters (*P*_12_, *P*_50_, *P*_88_ and slope) using an analysis of covariance (ANCOVA). A log transformation, when necessary, was applied to the predictive variables to deal with heteroscedasticity and/or non-normality (Zuur *et al.*, 2007). A post-hoc Tukey’s HSD test, from the R package Agricolae (Mendiburu, 2017), was used to test whether hydraulic parameters differ amongst species. To test the difference in *P*_50_ between the two Brassicaceae populations growing in contrasting environments (*H. incana* and *S. orientale*), we used linear mixed effects model, with the factor species as random effect, from the nlme R package (Pinheiro *et al.*, 2018).

We applied simple linear regressions to test for the relationship between *P*_50_, climate data and anatomical variables. A log transformation, when necessary, was performed on the predictive variables to deal with heteroscedasticity and/or non-normality (Zuur *et al.*, 2007).

In order to evaluate which anatomical variables explain embolism resistance, we performed a multiple linear regression with *P*_50_ as response variable and stem anatomical characters as predictive variables. We selected *a priori* the predictive variables using biological knowledge based on previously published studies in combination with a pairwise scatterplot to detect the presence of correlations and collinearities. Then, we conducted a variance inflation factor (VIF) analysis, keeping only variables with a VIF value <2 (Zuur *et al.*, 2010). Subsequently, we followed the model simplification removing each time the least significant variable, until all the remaining terms in the model were significant (Crawley, 2007). The regression or differences were considered significant if *P* < 0.05. Next, we calculated the hierarchical partitioning (Chevan and Sutherland, 1991) for the variables retained in the model in order to assess their relative importance to explain *P*_50_.

Independent *t*-tests were used to compare stem anatomical differences between the two populations of Brassicaceae species collected in contrasting environments.

To test whether differences in mean annual precipitation for each sampling site (P_R_) explained the combined variation of *P*_50_ and the anatomical characters, including also these characters that were not retained in the multiple regression analysis (the proportion of xylem fibre wall area per fibre area as observed in a cross-section, the thickness-to-span ratio of vessels and the hydraulically weighted vessel diameter), we performed a permutational multivariate analysis of variance (PERMANOVA). The anatomical characters and *P*_50_ are the response variables (rank transformed) and the mean annual precipitation is the predictive variable. PERMANOVA was performed using the adonis function in the Vegan R package (Oksanen *et al.*, 2015), based on Euclidean distances and 999 permutations. Later, a principal component analysis (PCA) was conducted using the function rda in the package Vegan, to observe simultaneously the relationships amongst the species, the main stem anatomical variables, the physiological variable (*P*_50_) and the mean annual precipitation (P_R_). We tested the relationship between some of the stem anatomical variables used in PCA with Pearson’s coefficient correlation.

All analyses were performed using R version 3.4.3 (R Core Team, 2017) in R Studio version 1.1.414 (R Studio Team, 2016). All the differences were considered significant when *P* was <0.05.

## RESULTS

### Interspecific and intraspecific vulnerability to xylem embolism in the herbaceous stems

**Fig. 1.**
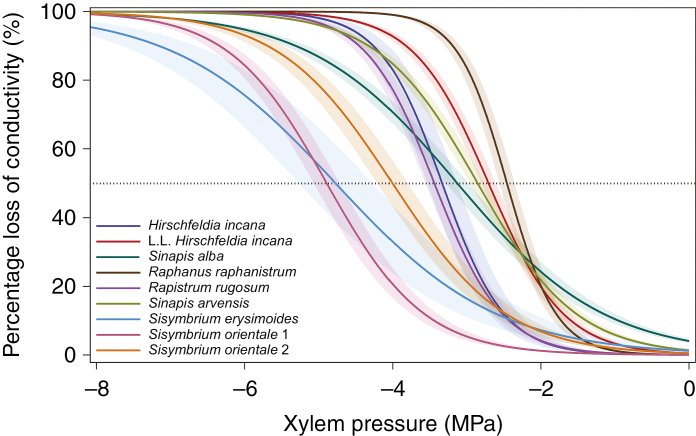
Mean vulnerability curves for each of the seven herbaceous Brassicaceae species studied native to different vegetation zones of Tenerife (Canary Islands), with reference to the sampling localities for *Hirschfeldia incana* and *Sisymbrium orientale*. Shaded bands represent *P*_50_ standard errors, and 50 % percentage loss of conductivity (PLC) is indicated by the horizontal dotted line. L.L. refers to the more humid population of *H. incana* collected in the city of La Laguna. The numbers 1 and 2 of *Sisymbrium orientale* refer to the populations collected in drier and more humid sites, respectively. See Supplementary Data Fig S1.

**Fig. 2.**
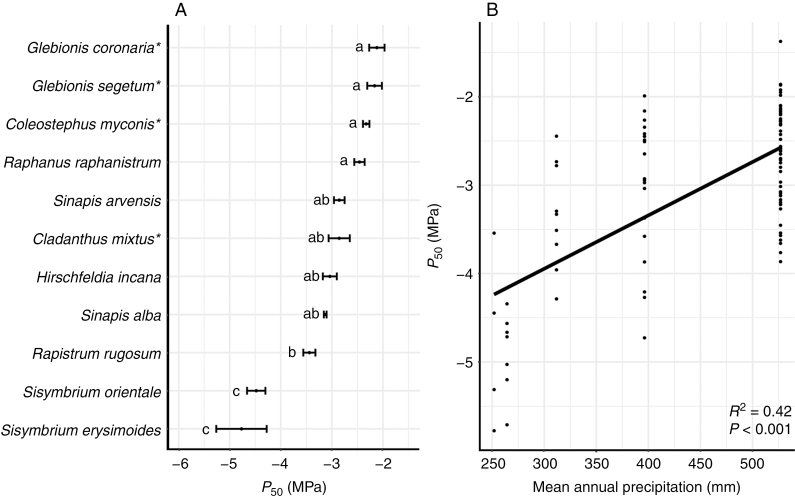
Range of stem *P*_50_ amongst seven herbaceous Brassicaceae and four Asteraceae (represented with an asterisk; data from Dória *et al.*, 2018) species from different vegetation zones in Tenerife (Canary Islands, Spain), and its relationship to mean annual precipitation. (A) Mean values of stem *P*_50_ of the herbaceous Brassicaceae and Asteraceae species studied. Standard errors are represented by bars. Different letters indicate differences between species at *P* < 0.05. (B) Relationship between *P*_50_ and mean annual precipitation at the individual level (on average six individuals per species). The adjusted *R*^2^ and level of significance is given.

The 11 herbaceous species studied show stem *P*_50_ values varying 2-fold from –2.1 MPa to –4.9 MPa (Figs 1 and 2A; see Dória *et al.*, 2018 for the vulnerability curves of Asteraceae species) (Supplementary Data Table S1). The range of stem *P*_50_ shows significant interspecific variation (*F* = 27.161, *P* < 0.001; Fig. 2A), with no interaction between species and mean annual precipitation (*F* = 2.948, *P* = 0.0901) (Supplementary Data Table S3). Species explain 70 % of the variance, regardless of the variation in mean annual precipitation for the sampling sites, while the mean annual precipitation (P_R_) explains 30 % of the variance, regardless of the variation in species (*F* = 16.689, *P* < 0.001; Fig. 2B) (Supplementary Data Table S3). Likewise, significant interspecific variations are also observed for *P*_88_ and *P*_12_ (*F* = 22.507, *P* < 0.001; *F* = 7.868, *P* < 0.001, respectively) with part of both variations explained by P_R_ (*F* = 6.506, *P* < 0.05; *F* = 4.439, *P* < 0.05 for *P*_88_ and *P*_12_, respectively). Variation in slope amongst the species studied is also significant (*F* = 4.940, *P* < 0.001), but the mean precipitation is not significant for this parameter (*F* = 0.138, *P* = 0.712).

**Fig. 3.**
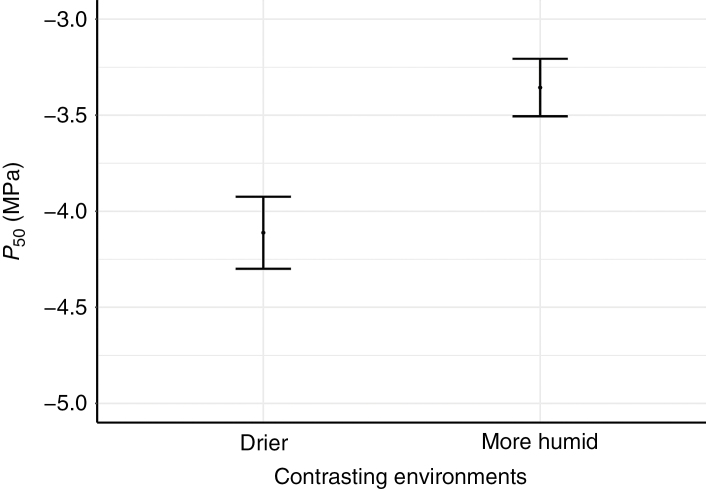
Intraspecific differences of mean stem *P*_50_ between the two populations of the Brassicaceae, *Hirschfeldia incana* and *Sisymbrium orientale*, collected in contrasting environments (*H. incana*: mean annual precipitation = 311.8 mm; aridity index = 0.39 for the drier site, and mean annual precipitation = 526.9 mm; aridity index = 0.68 for the more humid site. *S. orientale*: mean precipitation = 264.3 mm; aridity index = 0.34 for the drier site, and mean annual precipitation = 396.3 mm; aridity index = 0.53 for the more humid site.)

The two Brassicaceae populations of *H. incana* and *S. orientale* show significant intraspecific variation in *P*_50_ (*P* < 0.001, *F* = 17.6083), demonstrating that the contrasting environments are important to explain the intraspecific variation in *P*_50_ (Fig. 3). For *H. incana*, the drier site receives on average 311.8 mm of mean annual precipitation (aridity index = 0.39), while the more humid site receives on average 526.9 mm (aridity index = 0.68). For *S. orientale*, the drier site has on average 264.3 mm of mean annual precipitation, and the more humid site 396.3 mm for the same period (aridity index = 0.34 and 0.53, respectively) (Supplementary Data Fig. S1).

### Structure–function relationships in the herbaceous stems show correlation between embolism resistance and anatomy

**Fig. 4.**
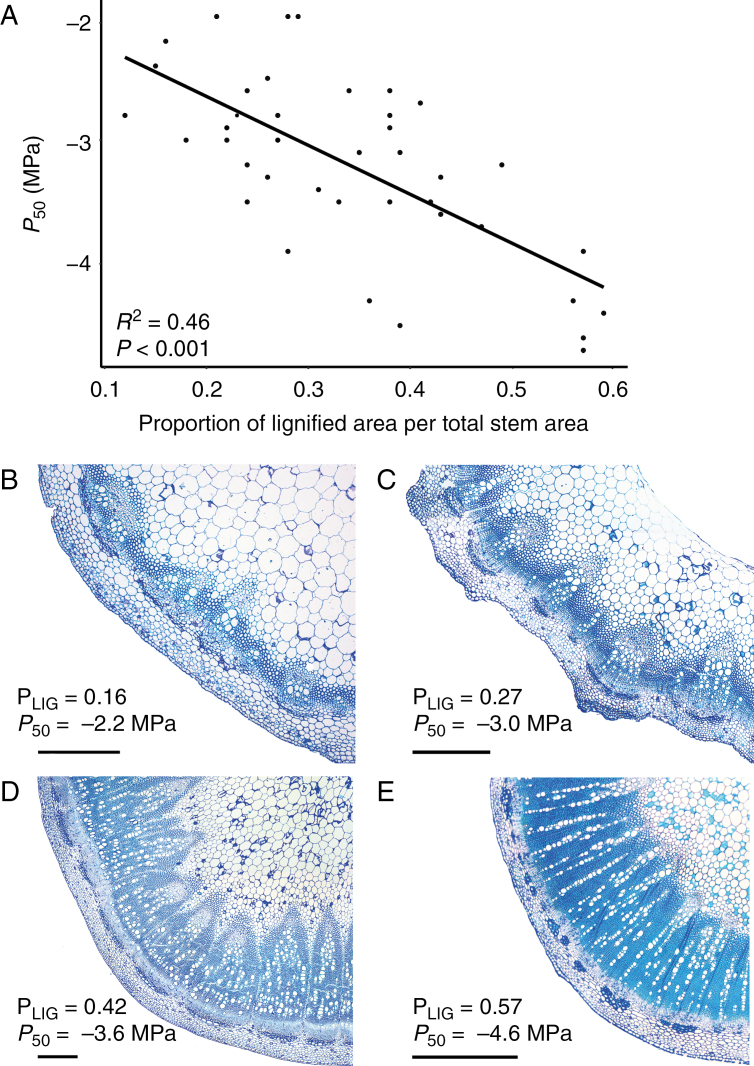
Relationships between stem *P*_50_ and the proportion of lignified area per total stem area (P_LIG_). (A) Linear regression between *P*_50_ and P_LIG_. The adjusted *R*^2^ and the level of significance are given. Each dot represents one individual (on average three individuals per species). (B–E) Light microscope images of cross-sections through the stem of Brassicaceae species showing an increase of P_LIG_ matching with an increase in embolism resistance. (B) *Raphanus raphanistrum*. (C) *Sinapis alba*. (D) *Rapistrum rugosum*. (E) *Sisymbrium orientale* from the drier sampling site. The scale bars represent 500 μm.

**Fig. 5.**
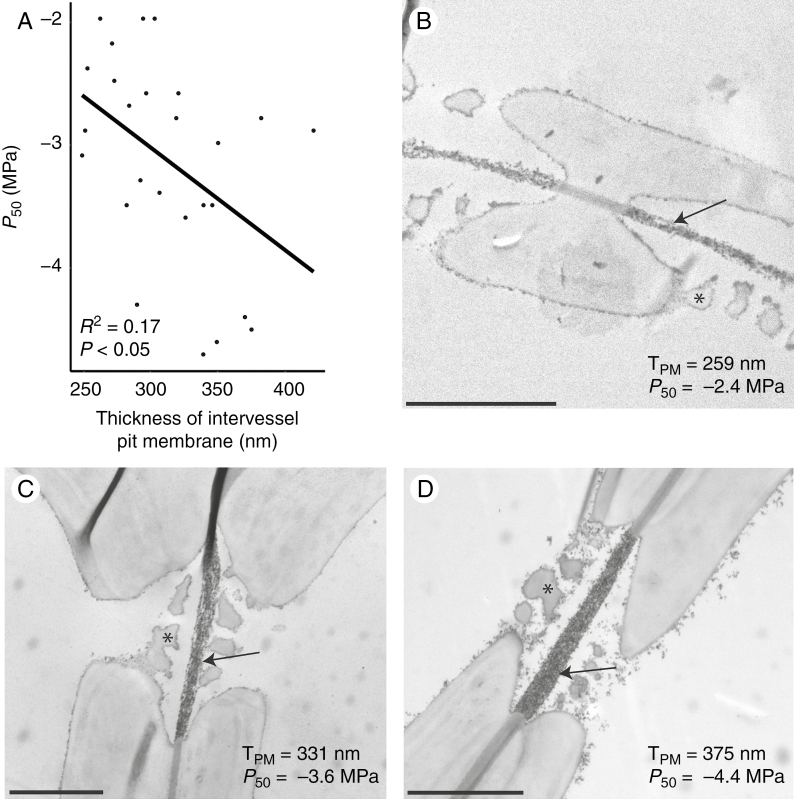
Relationships between stem *P*_50_ and thickness of the intervessel pit membrane (T_PM_). (A) Linear regression between *P*_50_ and T_PM_. The adjusted *R*^2^ and the level of significance are given. Each dot represents one individual (on average two individuals per species). (B–D) Transmission electron microscope images of intervessel pits of Brassicaceae species showing thicker pit membranes (arrows) in species that are more embolism resistant; all the herbaceous Brassicaceae species studied have vestures (asterisks). (B) *Raphanus raphanistrum*. (C) *Rapistrum rugosum*. (D) *Sisymbrium erysimoides*. Scale bars represent 2 μm.

The stem anatomical variables that best explain the variation in *P*_50_ are the proportion of lignified area per total stem area (P_LIG_; which is a measure of stem woodiness) (Fig. 4) and the thickness of the intervessel pit membrane (T_PM_) (Fig. 5) (*P* < 0.001; *R*^2^ = 0.6783) (Supplementary Data Tables S2 and S4). The *P*_50_–P_LIG_ relationship remains significant for the separate data sets (*P* < 0.001; *R*^2^ = 0.58 for Brassicaceae and *P* < 0.01; *R*^2^ = 0.48 for Asteraceae), while the *P*_50_–T_PM_ correlation disappears when analysing the Brassicaceae and Asteraceae data sets separately (*P* = 0.2164, *R*^2^ = 0.040 vs. *P* = 0.6175, *R*^2^ = –0.099, respectively). In addition, P_LIG_ is the main variable explaining 69 % of the *P*_50_ variation, while T_PM_ explains the remaining 31 % (Supplementary Data Tables S4).

**Fig. 6.**
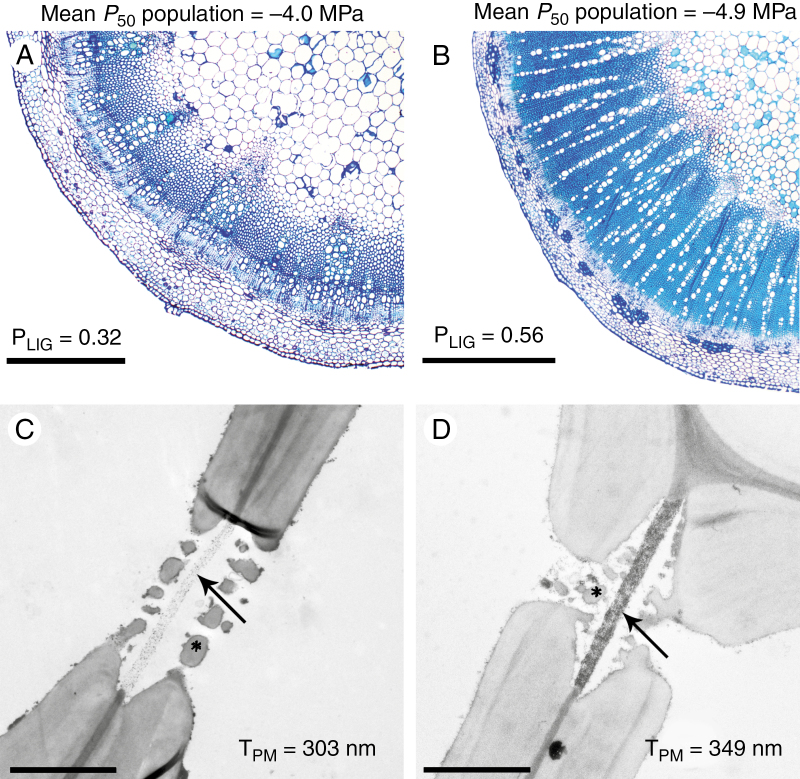
Intraspecific differences between two populations of *Sisymbrium orientale* growing in the more humid habitat (A, C) vs. the drier sampling site (B, D). (A, B) Light microscope image of cross-sections through the stems showing the population mean *P*_50_ values and proportion of lignified area per total stem area (P_LIG_). Scale bars represent 500 μm. (C, D) Transmission electron microscope images of intervessel pits showing the population mean of thickness of the intervessel pit membrane (T_PM_) (arrows). Vestures are marked with an asterisk. Scale bars represent 2 μm.

The *S. orientale* population growing in the drier sampling site shows a higher proportion of lignified area per total stem area (P_LIG_), thicker intervessel pit membranes (T_PM_) and thicker intervessel walls (T_VW_) than the population growing in the more humid sampling site (Fig. 6; Table 1) (Supplementary Data Table S2). No significant anatomical differences were found between the two populations of *H. incana* growing in contrasting environments.

**Table 1.** Stem anatomical variables that showed significant *t*-test differences between the two populations of *Sisymbrium orientale* growing in contrasting environments

Stem anatomical variable	Mean for *S. orientale* from the drier site	Mean for *S. orientale* from the more humid site	*t*-test (*P*-value)
Proportion of lignified area per total stem area	0.57	0.32	0.00763
Thickness of intervessel pit membrane (nm)	349.14	303.43	0.04231
Thickness of intervessel wall (μm)	3.70	3.31	0.01194

Mean annual precipitation for the drier site is 264.3 mm and for the more humid site is 396.3 mm; the aridity indexes are 0.34 and 0.53, respectively.

All Brassicaceae observed have vestured pits (Fig. 5B–D and 6C, D), while these are absent in the Asteraceae species. No differences in the level of vesturing are observed amongst the embolism-resistant vs. vulnerable Brassicaceae species.

### Relationship between mean precipitation (P_R_), stem anatomy and *P*_50_

The PERMANOVA test shows that the mean annual precipitation explains the variation in both stem anatomical characters and *P*_50_ (*F* = 3.8098, *R*^2^ = 0.14, *P* < 0.05) (Supplementary Data Table S5).

**Fig. 7.**
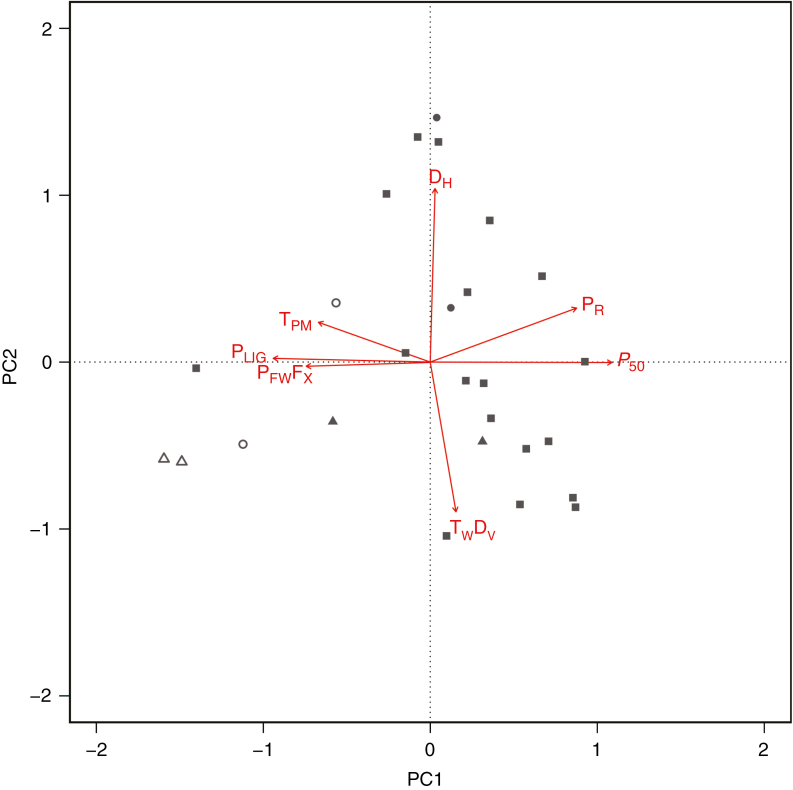
Principal component analysis of stem anatomical characters, mean annual precipitation and *P*_50_ on the first two axes. P_LIG_ = proportion of lignified area per total stem area as observed in a cross-section; P_FW_F_X_ = proportion of xylem fibre wall area per fibre area as observed in a cross-section; P_R_ = mean annual precipitation; T_W_D_V_ = thickness-to-span ratio of vessels; *P*_50_ = pressure inducing 50 % loss of hydraulic conductivity; D_H_ = hydraulically weighted vessel diameter; T_PM_ = thickness of intervessel pit membrane. Circles represent individuals of *H. incana* from humid (filled) and dry (open) sampling sites, while triangles refer to individuals of *S. orientale* from the humid (filled) and dry (open) sites. The squares represent the other individuals of Brassicaceae and Asteraceae studied.

When analysing the association amongst stem anatomical characters, mean annual precipitation and *P*_50_ using a PCA, the first axis of the PCA explains 40 % of the total variance observed, while the second axis explains 21 %. The first principal component has large positive associations with *P*_50_ and with mean annual precipitation (P_R_), and negative associations with the proportion of lignified area per total stem area as observed in a cross-section (P_LIG_), the proportion of xylem fibre wall area per fibre area as observed in a cross-section (P_FW_F_X_) and the thickness of intervessel pit membranes (T_PM_) (Fig. 7). Along this first axis, the proportion of xylem fibre wall per fibre is correlated with *P*_50_ (*P* < 0.01, *r* = –0.45). The second principal component has a large positive association with the hydraulically weighted vessel diameter (D_H_) and a negative association with the thickness-to-span ratio of vessels (T_W_D_V_). These two variables are negatively correlated with each other (*P* < 0.01, *r* = –0.51), but neither of them is correlated with embolism resistance (*P* = 0.7608, *r* = –0.0525; *P* = 0.5662, *r* = –0.0988). The thickness of the vessel is also not correlated with T_W_D_V_ (*P* = 0.2811, *r* = 0.1846). The individuals distributed at the right side of the multivariate PCA space are associated with less negative values of *P*_50_ and higher mean annual precipitation. Some of these individuals present higher values of the thickness-to-span ratio of vessels, while others have higher hydraulically weighted vessel diameters. In contrast, the individuals at the left side of the multivariate PCA space are associated with more negative values of *P*_50_, more pronounced lignification characters, thicker intervessel pit membranes and lower mean annual precipitation (Fig. 7).

Individuals of the two Brassicaceae populations of *H. incana* (represented by circles) and *S. orientale* (represented by triangles) occupy different areas of the multivariate space (Fig. 7). The individuals collected in drier sites (open circles for *H. incana* and open triangles for *S. orientale*) are associated with a higher degree of lignification characters, thicker intervessel pit membranes and lower values of mean annual precipitation (Fig. 7). The individuals collected in more humid sites (filled circles for *H. incana* and filled triangles for *S. orientale*) are associated with higher hydraulically weighted vessel diameter and higher values of the thickness-to-span ratio of vessels (Fig.7).

## DISCUSSION

Interspecific and intraspecific stem *P*_50_ variation across herbaceous eudicots is strongly linked to precipitation

Our data set, comprising 11 herbaceous species of Brassicaceae and Asteraceae from five different habitats in Tenerife with a mean annual precipitation from 252 to 527 mm, shows a 2-fold range of stem *P*_50_ values that match the precipitation values of the sampling sites: the most vulnerable species (*P*_50_ –2.1 MPa) was collected from wetter environments and the most resistant species (*P*_50_ –4.9 MPa) was sampled from drier vegetation types (Figs 1 and 2). The explanatory power of mean annual precipitation towards stem *P*_50_ supports the functional relevance of resistance to xylem embolism as an adaptive response to water deficit, as has been repeatedly demonstrated for woody trees (Maherali *et al.*, 2004; Blackman *et al.*, 2012; Choat *et al.*, 2012) and to a lesser extent also herbs (mainly grasses, Lens *et al.*, 2016). Likewise, the intraspecific (between-population) differences in stem *P*_50_ for both *S. orientale* and *H. incana* (Fig. 3) are also explained by mean annual precipitation: for both species, the more embolism-resistant populations occur in areas with less annual precipitation. This suggests that differences in habitat amongst herbaceous populations from the same species can increase the intraspecific plasticity in *P*_50_.

### Percentage of lignified area per total stem area (P_LIG_) outcompetes intervessel pit membrane (T_PM_) as the explanatory variable explaining variation in stem *P*_50_

The percentage of lignified area per total stem area (P_LIG_), which is mainly defined by the amount of woodiness in the herbaceous stems as observed in a cross-section, is the character that best explains the variation of embolism resistance in stems, with more lignified stems being more resistant to embolism (Fig. 4). Since the germination time of the herbaceous species on Tenerife does more or less converge after the arrival of the rains in autumn and winter, we believe that the differences in woodiness is species and/or niche specific rather than dependent on major differences in stem age between species. For example, the three species (*Raphanus raphanistrum*, *Sinapis arvensis* and the population of *Sisymbrium orientale* from the more humid area) collected in Vilaflor village (sampling site 4 of Supplementary Data Fig. S1) show a 2-fold difference in the degree of woodiness matching nicely with stem *P*_50_, despite the fact that these three populations occurred along the same road (Supplementary Data Tables S1 and S2). The relationship between characters related to higher stem lignification and higher absolute values of *P*_50_ has been recorded for different plant groups, both in woody (Hacke *et al.*, 2001; Jacobsen *et al.*, 2005; Jansen *et al.*, 2009; Pereira *et al.*, 2017) and in herbaceous lineages (Lens *et al*., 2012*b*, 2013, 2016; Tixier *et al.*, 2013) and in closely related woody lineages that are derived from herbaceous relatives (Dória *et al.*, 2018). Differences in the proportion of the lignified area in the stem are also found at the intraspecific level in this study, with the more resistant population of *S. orientale* showing thicker intervessel walls and higher P_LIG_ values compared with those of the more vulnerable population (Fig. 6; Table 1). The higher P_LIG_ values in the drier population could also be strengthened by the presumably earlier germination time in the area of El Escobonal (470 m asl), which is about 900 m lower than the colder (and wetter) site of Vilaflor (1400 m asl), making the stems of the drier (and lower) site older, enabling them to lignify more.

It is challenging to relate increased stem lignification functionally with embolism resistance, since most lignification characters do not directly influence embolism formation and spread in the 3-D network of angiosperm vessels. Indeed, the thickness of intervessel pit membranes (T_PM_) is more likely to affect the length of the tortuous and irregularly shaped pores that air–water menisci need to cross before air-seeding may occur, explaining the spread of embolism through intervessel pit membranes into adjacent conduits (Jansen *et al.*, 2009; Lens *et al*., 2011, 2013; Li *et al.*, 2016). Although the *P*_50_–T_PM_ relationship is confirmed in our herbaceous eudicot data set (Fig. 5), T_PM_ provides a much lower power to explain differences in *P*_50_ compared with the degree of woodiness as observed in a cross-section, calculated as the percentage of lignified area per total stem area (P_LIG_). This may seem surprising, but studies investigating the relationship between stem *P*_50_ and T_PM_ amongst herbaceous species are scarce and the functional relevance of T_PM_ in herbs might be less important compared with woody species. A few examples that suggest this poor *P*_50_–T_PM_ relationship in herbs are: the *P*_50_ – T_PM_ relationship disappears in our study when only including the Brassicaceae species; no link between *P*_50_ and T_PM_ was found in a grass data set based on four species with contrasting *P*_50_ values (Lens *et al.*, 2016); and a third study investigating closely related herbaceous and woody daisies showed that the *P*_50_–T_PM_ relationship was retrieved only when the herbaceous data set was combined with the woody data set (Dória *et al.*, 2018). Evidently, more work on stem *P*_50_ and additional anatomical measurements based on the same – properly fixated – herbaceous stems is needed to shed more light on the functional relevance of T_PM_ in herbs, which should in theory match the hydraulic importance of T_PM_ as observed in shrubs and trees (Li *et al.*, 2016).

Relationships between increased lignification and thicker intervessel pit membranes have been reported, which could explain the indirect correlation between higher lignification and higher embolism resistance (Jansen *et al.*, 2009; Li *et al.*, 2016; Dória *et al.* 2018). These findings are in accordance with our results for the two populations of *S. orientale* collected in contrasting environments (Table 1; Fig. 6): the more resistant population shows a higher proportion of lignified area in the stem, thicker intervessel wall, and thicker intervessel pit membranes. However, the T_PM_–lignification correlation disappears in our entire data set (including Asteraceae and Brassicaceae species), showing that increased lignification characters are not necessarily linked to thicker intervessel pit membranes.

### The mean precipitation explains both *P*_50_ and anatomical variation in stems of herbaceous eudicots

Mean annual precipitation explains both the variation in stem *P*_50_ and the variation in stem anatomical characters across the herbaceous species studied. It has been well documented that environmental factors influence *P*_50_ (Maherali *et al.*, 2004; Choat *et al.*, 2012; Trueba *et al.*, 2017) as well as anatomical traits (Carlquist, 1975; Baas *et al.*, 1983; Lens *et al.*, 2004; Dória *et al.*, 2016; O’Brien *et al.*, 2017). In our study, populations from drier sites show stems with more negative *P*_50_ values and more pronounced lignification, such as the proportion of lignified area per total stem area (a measure of the amount of woodiness) and the proportion of xylem fibre wall area per fibre area as observed in a cross-section. These characters are most associated with the first PCA axis (Fig. 7).

Our results show that the common pattern observed for woody species, i.e. a shift in rainfall patterns associated with survival and distribution of trees and shrubs (Engelbrecht *et al.*, 2007; Allen *et al.*, 2010; Trueba *et al.*, 2017), and drought-induced tree mortality associated with substantial loss of hydraulic conductivity across taxa and biomes (Adams *et al.*, 2017), is also true for herbaceous species (see also the first section of the Discussion). At the same time, different environment conditions also impact stem anatomical characters allowing plants to adapt to changing climates (Carlquist, 1975; Baas *et al.*, 1983; Martinez-Vilalta *et al.*, 2010; Kattge *et al.*, 2011).

Across woody trees, a lineage-specific sub-set of stem anatomical traits can be linked to drought-induced embolism resistance, such as increased wood density (linked to fibre wall thickness in angiosperms; Chave *et al.*, 2009; Zieminska *et al.*, 2013), increased thickness-to-span ratio of conduits (Hacke *et al.*, 2001; Bouche *et al.*, 2014), thicker intervessel pit membranes (Jansen *et al.*, 2009; Lens *et al.*, 2011; Li *et al.*, 2016; Dória *et al.*, 2018) and narrower vessel diameters (Poorter *et al.*, 2010; Hacke *et al.*, 2016; Olson *et al.*, 2018). Amongst herbaceous species, fragile stems also need to be reinforced by a suite of mechanical characters, as shown in our study: individuals occurring in drier areas show a higher degree of lignification/woodiness (P_LIG_) and thicker intervessel pit membranes (Fig. 7) (see previous section). The increment of cellular support against implosion is often cited as the reason for this hydraulic–mechanical trade-off, which can result from either an increase in vessel wall to lumen ratio (Hacke *et al.*, 2001; Jacobsen *et al.*, 2007; Cardoso *et al.*, 2018) or an increase in fibre matrix support (more and thicker walled xylem fibres) (Jacobsen *et al*., 2005, 2007; Pratt and Jacobsen, 2017; Dória *et al.*, 2018). For the herbaceous species studied here, we found the latter relationship, demonstrated by the correlation between a higher proportion of xylem fibre cell wall per fibre (P_FW_F_X_) and more negative *P*_50_. Both kinds of cellular reinforcements, due to either vessel wall reinforcements or a more pronounced surrounding fibre matrix, would result in increasing xylem density offering support against implosion. In accordance with this hydraulic–mechanical trade-off, collapse of xylem conduits was only observed in cells that lack a robust support of the fibre matrix, for instance in leaves (Cochard *et al.*, 2004; Brodribb and Holbrook, 2005; Zhang *et al.*, 2016) and in low-lignin stems of poplar mutants (Kitin *et al.*, 2010). Our study confirms that increasing the mechanical strength of fragile herbaceous stems using a suite of lignification characters may be highly relevant to acquire a higher level of embolism resistance.

Another aspect of the hydraulic–mechanical relationship in our data set is highlighted by the negative correlation between the thickness-to-span ratio of vessels (T_W_D_V_), determining the resistance to implosion of the conduit, and the hydraulically weighted vessel diameter (D_H_). Since there is a significant relationship between T_W_D_V_ and D_H_, but not between T_W_D_V_ and the thickness of the vessel wall (T_VW_), it can be concluded that vessel diameter impacts much more the variation of T_W_D_V_ than the thickness of vessel wall. It is known that larger vessel lumina increase hydraulic conductivity (Tyree and Zimmerman, 2002) and, because in our data set vessel wall thickness remains more or less the same, it gives rise to larger vessels that become mechanically weaker and potentially more vulnerable (Preston *et al.*, 2006; Zanne *et al.*, 2010; Pratt and Jacobsen, 2017). However, in our data set, *P*_50_ is not correlated with D_H_, with T_VW_ or with T_W_D_V_, meaning that the vessel diameter and thickness-to-span ratio of vessels do not impact embolism resistance in our herbaceous data set.

In conclusion, this study investigated structure–function relationships in stems of seven herbaceous Brassicaceae occurring in different vegetation zones across the island of Tenerife and merged the data set produced with a similar data set for herbaceous Asteraceae growing on the same island. The 2-fold difference in embolism resistance found here shows that stems of herbaceous eudicots are able to deal with a range of negative pressures inside xylem conduits, although the *P*_50_ range in woody trees remains considerably higher. In addition, mean annual precipitation is the major determinant influencing both embolism resistance and anatomical characters in the herbaceous stems, demonstrating the predictive value of both characters with respect to survival and distribution of herbs along environmental gradients. This improves our understanding of the evolutionary and ecological significance of embolism resistance in non-woody species. Our results also show that the degree of woodiness (P_LIG_) outcompetes the thickness of intervessel pit membranes (T_PM_) as the most powerful character determining embolism resistance in stems of herbaceous eudicots studied. This may question the hydraulic relevance of T_PM_ in herbs, although many more observations on embolism resistance and anatomical observations on herbaceous plants need to be carried out before a final conclusion can be reached.

## SUPPLEMENTARY DATA

Supplementary data are available online at https://academic.oup.com/aob and consist of the following. Figure S1: map of Tenerife with the five sampling sites, each corresponding to unique aridity indices. Table S1: hydraulic parameters of the herbaceous Brassicaceae species studied. Table S2: stem anatomical measurements of the herbaceous Brassicaceae species studied, along with the aridity indices and values for mean annual precipitation. Table S3: analysis of covariance of species and mean precipitation explaining the variance in *P*_50_ of the herbaceous Brassicaceae and Asteraceae species studied. Table S4: multiple regression model of anatomical features explaining the variance in *P*_50_ of the herbaceous Brassicaceae and Asteraceae species studied. Table S5: permutational multivariate analysis of variance of mean annual precipitation explaining the variance in *P*_50_ and in the main stem anatomical characters of the herbaceous Brassicaceae and Asteraceae species studied.

mcy233_suppl_Supplementary-Figure
