# Supplementary material for: Embolism resistance in stems of herbaceous Brassicaceae and Asteraceae is linked to differences in woodiness and precipitation
Source: Ann Bot. 2018 Dec 24;124(1):1–14. doi: 10.1093/aob/mcy233 (PMC6676380; doi:10.1093/aob/mcy233)
Supplement: mcy233_suppl_Supplementary-Figure [file mcy233_suppl_supplementary-figure.docx]

Fig. S1. Map of Tenerife with the five sampling sites, each corresponding to unique aridity indices (AI). 1 – San Andrés village, sampling site of *Sisymbrium erysimoides* (AI = 0.27); 2 – El Escobonal region, sampling site of the drier population of *Sisymbrium orientale* (AI = 0.34); 3 – Guímar municipality, sampling site of the drier population of *Hirschfeldia incana* (AI = 0.39); 4 – Vilaflor village, sampling site of the more humid population of *Sisymbrium orientale*, *Raphanus raphanistrum* and *Sinapis arvensis* (AI = 0.53); 5 – La Laguna town, sampling site of the more humid population of *Hirschfeldia incana*, *Rapistrum rugosum, Sinapis alba* and the four Asteraceae species *Cladanthus mixtus,* *Coleostephus myconis, Glebionis coronaria* and *Glebionis segetum* (AI = 0.68).


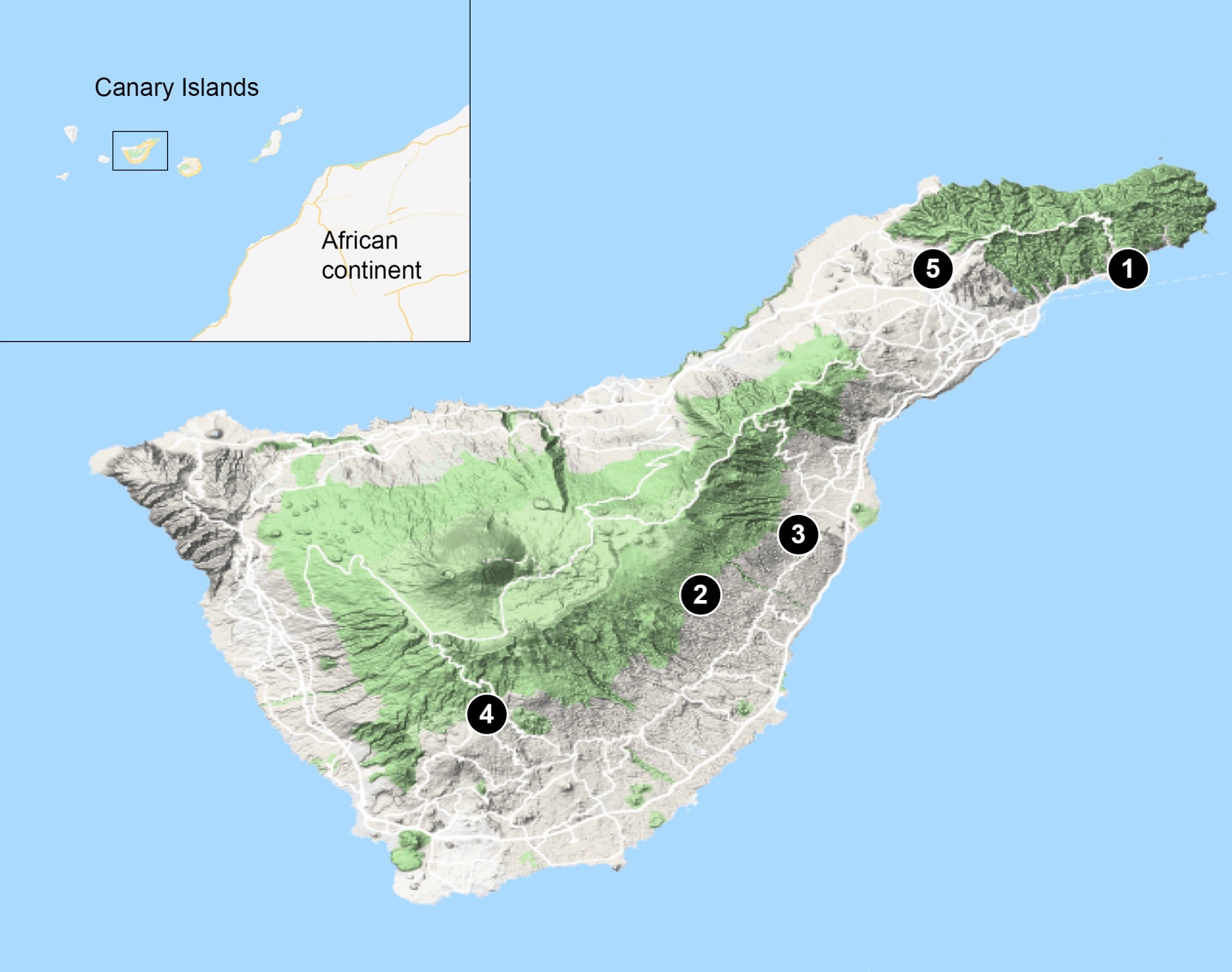


Table S1. Hydraulic parameters of the herbaceous Brassicaceae species studied. Mean value and standard error are given. For the Asteraceae species, see Dória *et al*. (2018).

| ***Species*** | ***P*_50_ (MPa)** | ***P*_12_ (MPa)** | ***P*_88_ (MPa)** | **Slope (%MPa^-1^)** | **K_MAX_ (m^2^MPa^-1^s^-1^)** |
| --- | --- | --- | --- | --- | --- |
| *Hirschfeldia incana*  (population from drier area) | -3.3±0.20 | -2.3±0.18 | -4.4±0.31 | 59.3±11.36 | 0.004062882±0.003005135 |
| Hirschfeldia incana  (population from more humid area) | -2.7±0.10 | -1.6±0.16 | -3.8±0.14 | 49.3±5.68 | 0.008323244±0.005518186 |
| *Sinapis alba* | -3.1±0.15 | -1.1±0.20 | -5.2±0.28 | 25.2±2.50 | 0.036192619±0.015321967 |
| *Sisymbrium erysimoides* | -4.8±0.49 | -2.3±0.10 | -7.2±0.95 | 23.0±4.98 | 4.19464E-05±2.04684E-05 |
| *Raphanus raphanistrum* | -2.5±0.10 | -1.7±0.15 | -3.2±0.11 | 75.0±9.33 | 7.17684E-05±1.90366E-05 |
| *Sisymbrium orientale* (population from drier area) | -4.9±0.17 | -2.9±0.50 | -6.9±0.36 | 37.9±10.82 | 0.00022239±2.71079E-05 |
| *Sisymbrium orientale* (population from more humid area) | -4.0±0.20 | -2.4±0.34 | -5.6±0.13 | 32.6±3.73 | 0.00031542±4.08823E-05 |
| *Rapistrum rugosum* | -3.4±0.12 | -2.2±0.25 | -4.7±0.28 | 54.3±15.29 | 0.080916805±0.032345281 |
| *Sinapis arvensis* | -2.9±0.10 | -1.5±0.14 | -4.2±0.11 | 37.4±1.77 | 0.015427343±0.007817781 |

*P*_50_ = pressure inducing 50% loss of hydraulic conductivity; *P*_12_ = pressure inducing 12% loss of hydraulic conductivity; *P*_88_ = pressure inducing 88% loss of hydraulic conductivity; Slope = indicator of the speed at which embolism affect the stem; K_MAX_ = maximum hydraulic conductance measured under xylem pressure close to zero.

Table S2: Stem anatomical measurements of the herbaceous Brassicaceae species studied, along with the aridity indices and values for mean annual precipitation. Mean values and standard deviation are given. For the Asteraceae species, see Dória *et al*. (2018).

| **Individuals** | **P_LIG_ (µm)** | **F_AP_** | **T_PM_ (nm)** | **D_H_ (µm)** | **P_FW_F_X_** | **T_VW_ (µm)** | **T_W_D_V_** | **AI** | **P_R_ (mm)** |
| --- | --- | --- | --- | --- | --- | --- | --- | --- | --- |
| *Hirschfeldia incana* 1  (Population from drier area) | 0.33 | 0.24±0.05 | 350.81±77.82 | 36.14 | 0.68±0.08 | 3.51±0.57 | 0.02±0.01 | 0.39 | 311.80 |
| *Hirschfeldia incana* 2  (Population from drier area) | 0.39 | 0.20±0.05 | 379.81±72.99 | 25.89 | 0.72±0.08 | 3.70±0.84 | 0.03±0.02 | 0.39 | 311.80 |
| *Hirschfeldia incana* 3  (Population from drier area) | 0.26 | 0.26±0.05 | - | 38.49 | 0.62±0.11 | 3.82±0.67 | 0.02±0.01 | 0.39 | 311.80 |
| *Hirschfeldia incana* 1 (Population from more humid area) | 0.27 | 0.27±0.04 | 386.98±70.91 | 43.58 | 0.67±0.09 | 4.37±0.83 | 0.01±0.006 | 0.68 | 526.90 |
| *Hirschfeldia incana* 2  (Population from more humid area) | 0.38 | 0.17±0.04 | 301.89±51.17 | 32.64 | 0.71±0.06 | 4.36±1.17 | 0.02±0.02 | 0.68 | 526.90 |
| *Hirschfeldia incana* 3  (Population from more humid area) | 0.35 | 0.20±0.03 | - | 37.42 | 0.84±0.07 | 4.06±0.82 | 0.02±0.02 | 0.68 | 526.90 |
| *Sinapis alba* 1 | 0.27 | 0.18±0.05 | 355.09±86.87 | 39.40 | 0.55±0.01 | 3.66±0.76 | 0.02±0.006 | 0.68 | 526.90 |
| *Sinapis alba* 2 | 0.38 | 0.16±0.04 | 344.31±58.05 | 42.45 | 0.54±0.11 | 3.80±0.60 | 0.01±0.004 | 0.68 | 526.90 |
| *Sinapis alba* 3 | 0.24 | 0.16±0.04 | - | 26.64 | 0.63±0.08 | 3.32±0.63 | 0.03±0.03 | 0.68 | 526.90 |
| *Sisymbrium erysimoides* 1 | 0.59 | 0.19±0.04 | 374.95±92.04 | 29.64 | 0.64±0.08 | 4.19±1.00 | 0.02±0.02 | 0.27 | 251.90 |
| *Sisymbrium erysimoides* 2 | 0.24 | 0.23±0.06 | - | 19.28 | 0.62±0.08 | 3.23±0.83 | 0.04±0.03 | 0.27 | 251.90 |
| *Sisymbrium erysimoides* 3 | 0.20 | 0.26±0.06 | 372.97±83.35 | 18.74 | 0.65±0.08 | 3.37±0.72 | 0.06±0.04 | 0.27 | 251.90 |
| *Raphanus raphanistrum* 1 | 0.15 | 0.25±0.07 | 258.46±56.18 | 18.27 | 0.52±0.10 | 2.51±0.44 | 0.02±0.01 | 0.53 | 396.30 |
| *Raphanus raphanistrum* 2 | 0.16 | 0.17±0.05 | 276.65±57.50 | 39.87 | 0.61±0.07 | 3.87±0.82 | 0.02±0.01 | 0.53 | 396.30 |
| *Raphanus raphanistrum* 3 | 0.18 | 0.28±0.06 | - | 25.39 | 0.47±0.01 | 2.72±0.52 | 0.03±0.02 | 0.53 | 396.30 |
| *Sisymbrium orientale* 1  (Population from drier area) | 0.57 | 0.17±0.03 | 354.17±68.21 | 26.25 | 0.78±0.12 | 3.75±0.66 | 0.03±0.02 | 0.34 | 264.30 |
| *Sisymbrium orientale* 2 (Population from drier area) | 0.57 | 0.18±0.03 | 344.11±54.05 | 26.29 | 0.73±0.08 | 3.63±0.66 | 0.03±0.02 | 0.34 | 264.30 |
| *Sisymbrium orientale* 3  (Population from drier area) | 0.56 | 0.18±0.04 | - | 23.51 | 0.76±0.07 | 3.73±0.75 | 0.03±0.01 | 0.34 | 264.30 |
| *Sisymbrium orientale* 1  (Population from more humid area) | 0.36 | 0.19±0.06 | 295.12±87.16 | 29.47 | 0.73±0.07 | 3.46±0.93 | 0.03±0.02 | 0.53 | 396.30 |
| *Sisymbrium orientale* 2  (Population from more humid area) | 0.31 | 0.21±0.05 | 311.74±67.95 | 21.59 | 0.42±0.11 | 3.18±0.57 | 0.02±0.007 | 0.53 | 396.30 |
| *Sisymbrium orientale* 3  (Population from more humid area) | 0.28 | 0.12±0.03 | - | 24.37 | 0.69±0.08 | 3.3±0.64 | 0.02±0.01 | 0.53 | 396.30 |
| *Rapistrum rugosum* 1 | 0.42 | 0.10±0.03 | 287.42±42.02 | 45.12 | 0.66±0.07 | 4.39±0.88 | 0.01±0.007 | 0.68 | 526.90 |
| *Rapistrum rugosum* 2 | 0.43 | 0.12±0.02 | 330.99±133.3 | 42.85 | 0.67±0.08 | 4.67±1.02 | 0.02±0.01 | 0.68 | 526.90 |
| *Rapistrum rugosum* 3 | 0.49 | 0.13±0.03 | - | 50.54 | 0.66±0.08 | 4.4±0.98 | 0.01±0.01 | 0.68 | 526.90 |
| *Sinapis arvensis* 1 | 0.22 | 0.20±0.06 | 425.72±84.18 | 26.54 | 0.61±0.07 | 3.43±0.60 | 0.02±0.01 | 0.53 | 396.30 |
| *Sinapis arvensis* 2 | 0.24 | 0.29±0.05 | 325.72±91.35 | 27.16 | 0.57±0.08 | 3.75±0.75 | 0.02±0.01 | 0.53 | 396.30 |
| *Sinapis arvensis* 3 | 0.22 | 0.19±0.04 | - | 25.17 | 0.58±0.08 | 3.66±0.67 | 0.02±0.01 | 0.53 | 396.30 |

P_LIG_ = proportion of lignified area per total stem area; F_AP_ = intervessel pit aperture fraction (pit aperture area / bordered pit area); T_PM_ = thickness of the intervessel pit membrane; D_H_ = hydraulically weighted vessel diameter; P_FW_F_X_ = proportion of xylem fiber wall area per fiber area as observed in a cross section; T_VW_ = thickness of the vessel wall; T_W_D_V_ = thickness-to-span ratio of vessels; AI = aridity index; P_R_ = mean annual precipitation.

Table S3: Analysis of covariance of species and mean precipitation explaining the variance in *P*_50_ of the herbaceous Brassicaceae and Asteraceae species studied.

| **Source of variation** | **Degrees of freedom** | **Sum of squares** | **Mean of Squares** | **F Value** | **P value** | **Hierarchical Partitioning** |
| --- | --- | --- | --- | --- | --- | --- |
| Species | 10 | 58.02 | 5.802 | 27.161 | <2e-16 | 70.15 |
| Precipitation | 1 | 3.56 | 3.565 | 16.689 | 0.000109 | 29.85 |
| Species: Precipitation | 1 | 0.63 | 0.630 | 2.948 | 0.090109 | --- |
| Residuals | 75 | 16.02 | 0.214 | --- | --- | --- |

Table S4. Multiple regression model of anatomical features explaining the variance in *P*_50_ of the herbaceous Brassicaceae and Asteraceae species studied.

| **Source of variation** | **Parameter estimate** | **SE** | **t-value** | **P value** | **Hierarchical Partitioning** |
| --- | --- | --- | --- | --- | --- |
| Intercept | 0.3675 | 0.6893 | 0.533 | 0.5991 | --- |
| P_LIG_ | -5.0281 | 0.8040 | -6.254 | 2.21e-06 | 69.2903 |
| T_PM_ | -0.0056 | 0.0021 | -2.620 | 0.0153 | 30.7097 |

P_LIG_ = the proportion of lignified area per total stem area; T_PM_ = thickness of intervessel pit membranes.

Table S5. Permutational multivariate analysis of variance of mean annual precipitation explaining the variance in *P*_50_ and in the main stem anatomical characters of the herbaceous Brassicaceae and Asteraceae species studied.

| **Source of variation** | **Degrees of freedom** | **Sum of Squares** | **Mean of Squares** | **F. Model** | **R^2^** | **P value** |
| --- | --- | --- | --- | --- | --- | --- |
| Precipitation | 1 | 0.2026 | 0.2026 | 3.8098 | 0.137 | 0.017 |
| Residuals | 24 | 1.2763 | 0.05318 | --- | 0.863 | --- |
| Total | 25 | 1.4789 | --- | --- | 1.000 | --- |
